# Protective Effect of Jiang Tang Xiao Ke Granules against Skeletal Muscle IR via Activation of the AMPK/SIRT1/PGC-1*α* Signaling Pathway

**DOI:** 10.1155/2021/5566053

**Published:** 2021-07-03

**Authors:** Ying Bai, Jiacheng Zuo, Xin Fang, Rufeng Ma, Tian Tian, Fangfang Mo, Qianqian Mu, Yi Zhang, Na Yu, Xueli Bao, Dongwei Zhang, Sihua Gao, Dandan Zhao

**Affiliations:** ^1^College of Traditional Chinese Medicine, Beijing University of Chinese Medicine, Beijing, China; ^2^Beijing University of Chinese Medicine Affiliated Third Hospital, Beijing, China; ^3^Dongzhimen Hospital of Beijing University of Chinese Medicine, Beijing, China; ^4^School of City Management, Beijing Open University, Beijing, China; ^5^Educational Office, Beijing Tiantan Hospital, Capital Medical University, Beijing, China

## Abstract

The Jiang Tang Xiao Ke (JTXK) granule is a classic Chinese herbal formula that has been put into clinical use in the treatment of type 2 diabetes mellitus for decades. However, whether its ability to ameliorate skeletal muscle insulin resistance (IR) is through modulation of the AMPK/SIRT1/PGC-1*α* signaling pathway remains unknown. Therefore, we aimed to investigate the effects of JTXK granules on IR in skeletal muscle of high-fat diet-induced diabetic mice and C2C12 cells and analyze the underlying mechanisms. In the present study, we showed that JTXK granules attenuated body weight gain, reduced body fat mass, improved body lean mass, and enhanced muscle performance of diabetic mice. JTXK granules also improved glucose metabolism and skeletal muscle insulin sensitivity and partially reversed abnormal serum lipid levels, which might be related to the regulation of the AMPK/SIRT1/PGC-1*α* pathway, both in skeletal muscle tissue of diabetic mice and in C2C12 cells. Furthermore, drug-containing serum of JTXK granules was capable of enhancing glucose uptake and mitochondrial respiration in C2C12 cells, and AMPK*α* was proven to be closely involved in this process. Taken together, these results suggest that the JTXK granule ameliorates skeletal muscle IR through activation of the AMPK/SIRT1/PGC-1*α* signaling pathway, which offers a novel perspective of this formula to combat IR-related metabolic diseases.

## 1. Introduction

Insulin resistance (IR) is a heterogeneous metabolic defect that primarily refers to the abnormality of insulin-mediated glucose disposal [1]. IR is not only the main characteristic of obesity or type 2 diabetes mellitus (T2DM) but also the promoter of these metabolic diseases [2]. Skeletal muscle, accounting for the largest portion of insulin-mediated glucose uptake, utilization, and storage, is crucial in metabolic disorders [3, 4]. Enormous evidence has shown that mitochondrial dysfunction in skeletal muscle tissue plays an essential role in impaired insulin signaling. A reduced rate of mitochondrial ATP synthesis was observed in skeletal muscle of obese patients, prediabetic patients, and offspring of diabetic patients [5, 6]. Long-term hyperglycemia and hyperlipidemia lead to mitochondrial impairment [7], and mitochondrial dysfunction in turn will aggravate glucolipid metabolic disorder [8]. Therefore, maintaining the functional integrity of skeletal muscle mitochondria is considered an important approach to improve insulin sensitivity and glucolipid metabolism [9, 10].

Activation of adenosine monophosphate-activated protein kinase (AMPK) in response to insulin can boost the translocation of GLUT4 from the cytoplasm to the membrane and promote glucose uptake, transportation, and utilization [11]. Besides that, AMPK activation can also improve fatty acid oxidation, reduce ectopic lipid deposition in skeletal muscle cells, and thus increase insulin sensitivity [12]. In addition, AMPK promotes mitochondrial synthesis by directly phosphorylating peroxisome proliferator-activated receptor coactivator 1 *α* (PGC-1*α*). PGC-1*α* is known to increase gene expressions that are related to long-chain fatty acid oxidation, mitochondrial DNA replication, and cellular oxidative metabolism [13]. Meanwhile, sirtuin 1 (SIRT1) is an important energy metabolism monitor with NAD^+^-dependent deacetylase activity, and it has been proven to interact with PGC-1*α* [14]. By activating the AMPK/SIRT1/PGC-1*α* axis, mitochondrial synthesis and oxidative phosphorylation can be promoted, and high-fat diet- (HFD) induced obesity and IR thus can be retarded.

The Jiang Tang Xiao Ke (JTXK) granule, composed of *Rehmannia glutinosa* (Gaertn.) DC. (Dihuang), *Panax ginseng* C.A.Mey. (Renshen), *Coptis chinensis* Franch. (Huanglian), *Salvia miltiorrhiza* Bunge (Danshen), and so on, is a classic formula from traditional Chinese medicine. Our previous studies have shown that JTXK granules exhibited an overt therapeutic effect in the management of diabetes through the improvement of glucolipid metabolism, protection of islet ßeta-cells, and inhibition of oxidative stress and inflammatory reactions in various organs and tissues [15–18]. There are studies supporting that the main active ingredients from this herbal formula, for example, berberine and ginsenoside Rb1, are capable of activating AMPK signaling [19, 20]. However, whether and how JTXK granule ameliorates skeletal muscle IR is still not clear. Therefore, we attempted to investigate the role of JTXK granules in the control of skeletal muscle IR in HFD-induced diabetic mice and C2C12 cells, as well as their influence on the AMPK/SIRT1/PGC-1*α* signaling pathway.

## 2. Materials and Methods

### 2.1. Reagents and Equipment

JTXK granule was produced and quality-controlled as previously reported [17]. The fingerprint of this granule that illustrated the main ingredients is shown in Supplementary Figure 1 (S1). C2C12 cells were obtained from the National Infrastructure of Cell Line Resource (Beijing, China). The blood lipid kits were purchased from Nanjing Jiancheng Bioengineering Institute (Nanjing, China). Equipment used in the study includes the NMR animal body composition analyzer from Shanghai Newmai Electronic Technology Co., Ltd. (Shanghai, China), as well as the Hitachi 7080 automatic biochemical analyzer (Tokyo, Japan). Antibodies against AMPK*α*, SIRT1, PGC-1*α*, PPAR*α*, and UCP3 (Cat #: ab32047, ab110304, ab54481, ab215270, and ab180643) were purchased from Abcam (Cambridge, UK); antibodies against CPT1 and Na,K-ATPase (ab12252; 3010S) were from Cell Signaling Technology (Danvers, USA); and antibodies against GLUT4 and Lamin B1 (66846-1-lg; 66095-1-lg) were from Proteintech (Rosemont, USA). All other materials are commercially available.

### 2.2. Animal Models and Experimental Design

Male 8-week-old C57BL6/J mice were purchased from Beijing Sibeifu Animal Technology Co., Ltd. (Beijing, China). Mice were kept on an ad libitum basis and allowed free access to tap water and feed. All the mice were housed in the animal laboratory with a barrier environment in Beijing University of Chinese Medicine (certification number SCXK (Jing) 2016-0002) with a temperature of 25°C and humidity of 55 ± 5%. The protocols were approved by the Animal Care Committee of Beijing University of Chinese Medicine (Beijing, China).

**Figure 1:**
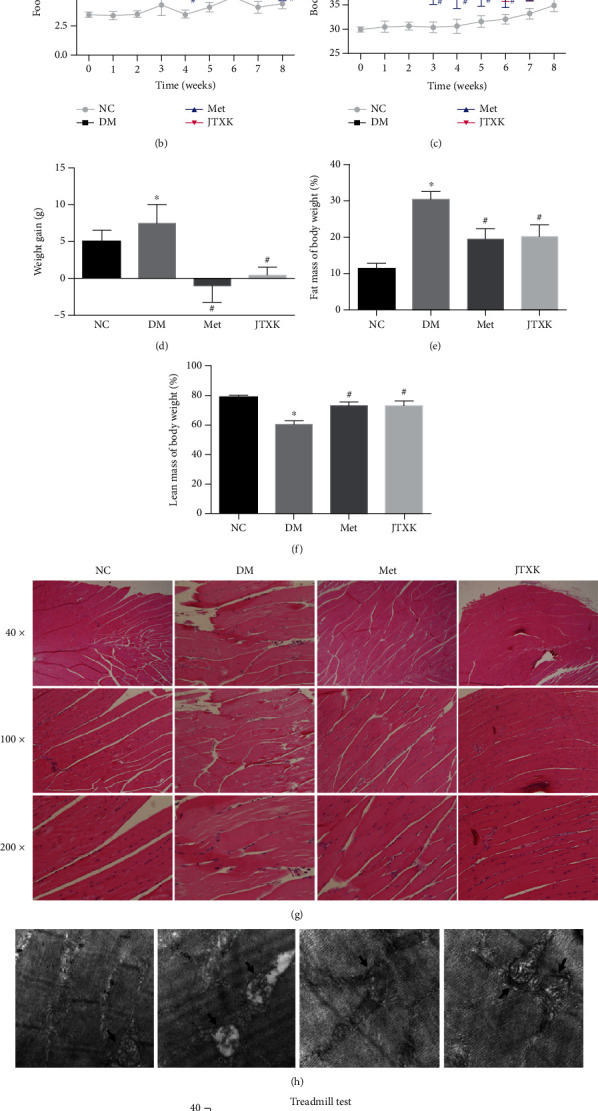
Effect of JTXK granules on HFD-fed diabetic mice. (a) Experimental design of the present study. (b) Food intake of mice in each group. (c, d) Body weight alteration. (e) Body fat rate and (f) body lean rate of mice after the intervention. (g) H&E staining and (h) electron microscopy of skeletal muscle tissue. (i) Treadmill test results of the mice in each group. NC, DM, Met, and JTXK mean the normal control, diabetes, metformin, and Jiang Tang Xiao Ke granule groups, respectively. Data were presented as mean ± SD. *n* = 8 in each group. ^∗^*P* < 0.05, compared with the NC group. ^#^*P* < 0.05, compared with the DM group.

After one week of adaptive feeding, mice were randomly divided into the normal group (NC group, *n* = 8) and HFD-fed group (*n* = 32). Mice in the normal group were fed the standard chow diet (AIN-96G feed from Sibeifu Bioscience Co., Ltd., Beijing, China), while their counterparts received HFD feeding, of which 45% of calories are from fat (MD12032, Jiangsu Hengrui Medicine Co., Ltd., Jiangsu, China) during the entire experiment. The diabetic model induction process lasted 12 weeks. After 12 weeks of feeding, mice whose body weight gain was 20% heavier than the average weight of the normal group with fasting blood glucose higher than 7.0 mmol/L were considered diabetic mice [21]. Then, the eligible diabetic mice were randomly divided into the model group (DM group), metformin group (Met group), and JTXK group with 8 mice in each. Mice in the metformin group were administrated with metformin (100 mg/kg) by gavage; mice in the JTXK group were given JTXK granules dissolved in sterilized water (1.75 g/kg); mice in the normal and DM groups received the same amount of sterile water. During the treatment, body weight and food intake were recorded once a week. After 8 weeks of intervention, the body composition of all mice was assessed by the NMR animal body composition analyzer. Then, all mice were sacrificed after anesthesia with 4% formaldehyde with their blood sample and skeletal muscle tissue collected for further analysis. The experimental design is shown in Figure 1(a).

### 2.3. Glucose and Insulin Tolerance Test

The oral glucose tolerance test (OGTT) was performed after the mice were fasted for 8 h from 7:00 a.m. to 3:00 p.m. Blood glucose levels were assessed before and 30, 60, 90, and 120 min after intragastric administration of glucose (2 g/kg) to the mice. For the insulin tolerance test (ITT), mice were firstly fasted for 5 h from 8:00 a.m. to 1:00 p.m., then were injected intraperitoneally with insulin (0.5 U/kg). Blood glucose levels were measured before and 30, 60, 90, and 120 min after injection.

### 2.4. Treadmill Test

A treadmill test was used to evaluate exercise capacity and endurance. Before the formal test, mice in each group received a 6-day treadmill adaption training, which was to set the speed to 10 m/min and the running platform to 5° and allow mice to run for 5 min. When the formal test was performed, set the treadmill to 5° and the speed to 20 m/min [20]. The exhaustion standard included the following: continuous drop into the powder grip with shock, resting in the abdomen position, being always in the last third of the runway, being short of breath, and unable to run on the treadmill for 10 s despite mechanical prodding. The exhaustion time of each mouse was recorded for analysis.

### 2.5. Biochemical Analysis

Serum triglyceride (TG), total cholesterol (TC), high-density lipoprotein (HDL), and low-density lipoprotein (LDL) were determined using the commercial assay kits from Nanjing Jiancheng Bioengineering Institute (Nanjing, China). All the assays were conducted in accordance with the manufacturer's instructions. Absorbance values of these measurements were detected by the automatic biochemical analyzer.

### 2.6. Skeletal Muscle Tissue Hematoxylin and Eosin Staining

The skeletal muscles were exposed, and then the gastrocnemius muscles from the hind limb were collected. The skeletal muscle tissue was fixed in a 4% neutral formaldehyde buffer for 24 h and then embedded in paraffin. Next, the embedded paraffin blocks were cut into 4 *μ*m sections and stained with hematoxylin and eosin according to the set routine. The optical microscope (Olympus, Japan) was used to observe the histopathological changes in muscular tissue.

### 2.7. Transmission Electron Microscopy (TEM)

Skeletal tissue was immediately rinsed in PBS after removing from the mice. The tissue was diced into 2 mm cubes and fixed with 4% glutaraldehyde and 0.2% tannic acid in PBS overnight. Samples were washed with PBS and postfixed in 1% osmium tetroxide for 2 h. Samples were then dehydrated through a graded series of acetone and embedded in SPI-Pon 812 resin (SPI Co., USA) 24 h at 36°C, 48°C, and 60°C, respectively. Embedded tissue was cut into 70 nm sections and stained with uranyl acetate (30 min), followed by 0.2% lead citrate (30 min). Images were photographed under an electron microscope (Hitachi H-7650, Japan).

### 2.8. Cell Culture and Experimental Design

C2C12 cells were cultured in DMEM containing 10% fetal bovine serum (FBS). All the cells were cultured at 37°C with 95% humidity and 5% CO_2_. Cells were passaged every 2-3 days. When cells grew to 80% confluence, the medium was replaced by high-glucose (25 mM) DMEM containing 2% horse serum to induce differentiation. Differentiation media were replenished every other day by removing 75% of the cell medium, followed by adding the fresh medium. Then, the differentiated C2C12 myotubes were treated with DMEM containing 0.4 mmol/L of palmitic acid and 1% FBS for 24 h to induce IR. For the treatment, the C2C12 myotube cells were incubated in the medium containing DCS of JTXK or control serum for 48 h.

### 2.9. Preparation of Drug-Containing Serum of JTXK

The drug-containing serum (DCS) of JTXK was obtained following our previous procedures [22]. Specifically, we administrated JTXK granules to rats (10.7 g/kg BW) by gavage for 3 consecutive days. And on the third day, rats were sacrificed and blood samples were taken and centrifuged for collecting the DCS. The control rats were given the same amount of water to provide control serum.

### 2.10. Construction of the AMPK*α* Knockdown Cell Line

Generation of the AMPK*α* knockdown cell line was accomplished by Genloci Biotechnologies, Inc. (Jiangsu, China). The sequence of the siRNA oligo specific for AMPK*α* (Gene ID: 105787) was GTTGGATTTCCGTAGTATTAT. Cells were suspended in a sterile tube, centrifuged at 1000 rpm for 4 min, and added with DPBS. And then, 30 *μ*g of pEGFP-N1 plasmid with siRNA was added to the system and delivered to the cells by electroporation at 600 V (30 ms, 1 pulse). The transfected cells were cultured in DMEM for 48 h and then transferred into a medium containing 2 *μ*g/mL Puro. Then, limiting dilution analysis was applied to screen out positive clones and RT-PCR was conducted to confirm the transfection rate.

### 2.11. Glucose Consumption Assay

The glucose oxidase method was used to examine the glucose levels in the culture medium of the C2C12 cells. According to the manufacturer's instructions, 5 *μ*L of the supernatant or different concentrations of the glucose standard solution were added into a 195 *μ*L working solution. The absorbance was determined at 570 nm after incubation for 20 min at 37°C. The glucose consumption was calculated by the glucose concentration of blank wells subtracted from that of cell-plated wells.

### 2.12. Mitochondrial Function Evaluation

Mitochondrial function was evaluated by measuring the oxygen consumption rate on the Seahorse XF Extracellular Flux Analyzer (Seahorse Bioscience, USA) according to the manufacturer's protocol. The C2C12 cells were treated with the JTXK drug-containing serum or control vehicle in XF24 analyzer microplates. 48 h later, the medium was changed to an XF basic medium supplemented with 11 mM glucose, 4 mM glutamine, and 2 mM pyruvate. Then, the cells were incubated with the sequential addition of 1 *μ*M oligomycin, 5 *μ*M carbonyl cyanide-(trifluoromethoxy)phenylhydrazone, and 0.5 *μ*M rotenone/antimycin A. The mitochondrial respiratory parameters, such as basal respiration, ATP production, maximum respiration, and spare respiratory capacity, were calculated.

### 2.13. Western Blotting

Proteins were extracted from skeletal muscle tissue and C2C12 cells with a precool RIPA lysis buffer containing 1% PMSF. After centrifugation, the total protein was extracted and the concentration was measured according to the BCA kit. Equal amounts of total protein per sample were subjected via SDS-PAGE separation gel and concentration gel and transferred to polyvinylidene fluoride (PVDF) membranes using a wet blotting system (Bio-Rad Laboratories, Inc., USA). Membranes were blocked with 5% skim milk dissolved in Tris-buffered saline and incubated with diluted AMPK*α*, CPT1, PGC-1*α*, PPAR*α*, SIRT1, UCP3, and GLUT4 (1 : 1000, 5% skim milk diluted) at 4°C overnight. On the following day, the membrane was incubated for 1.5 h with the horseradish peroxidase- (HRP-) conjugated secondary antibody (1 : 5000 diluted) at room temperature. Then, the membranes were incubated with high-sensitivity ECL luminous liquid (Proteintech Biotechnology, USA), and images were captured with Azure Biosystems, Inc., USA. Densitometry analyses of immunoblots were performed with the ImageJ software package.

### 2.14. Real-Time PCR (RT-PCR)

Total RNA was isolated from skeletal muscle and C2C12 cells with the TRIzol reagent according to the manufacturer's instructions. cDNA was synthesized using a RevertAid First Strand cDNA Synthesis Kit (Thermo Fisher Scientific, USA). RT-PCR was performed on a StepOne™ RT-PCR System (Applied Biosystems, USA) using the SYBR Green Master Mix (Invitrogen, USA). The amplification conditions were set as follows: predenaturation at 95°C for 10 min, followed by 40 cycles of amplification (95°C for 15 s, 60°C for 60 s). For each sample, PCR reactions were run in triplicate. And for each group, there were six samples. The gene-specific primers are shown in Table 1. Calculations were performed by a comparative method (such as 2^-*ΔΔ*CT^).

### 2.15. Statistical Analysis

All data were presented as mean ± standard deviation (SD). Datasets with more than two groups to be compared were analyzed using one-way analysis of variance (ANOVA) and Tukey's post hoc multiple comparison test. For statistical analyses of groups comparing two variables, two-way ANOVA was conducted, followed by Tukey's post hoc multiple comparison test. *P* < 0.05 was considered statistically significant. All the figures and statistical analyses were generated on GraphPad Prism 7 (GraphPad Software, USA).

## 3. Results

### 3.1. JTXK Granules Attenuated Body Weight Gain and Improved Skeletal Muscle Content and Performance

Twelve weeks of HFD feeding caused significant overeating of obese diabetic mice, while treatment with metformin or JTXK significantly reduced food intake (Figure 1(b)). As a consequence, mice in the metformin and JTXK groups exhibited significant weight loss compared with DM group mice after corresponding treatment (Figures 1(c) and 1(d)). The results of body composition showed that after 8 weeks of treatment, mice in the DM group showed a significantly higher body fat rate and less body lean mass than mice in the normal control group. Body fat rates of mice in the Met and JTXK groups, which were 19.50% and 20.29%, respectively, were significantly lower than that of DM mice (30.46%) (Figure 1(e), *P* < 0.05). Meanwhile, in comparison with DM mice, metformin and JTXK granules both improved the body lean mass of diabetic mice (Figure 1(f), *P* < 0.05). Moreover, according to results from the treadmill test, a higher body fat rate and less body lean mass accompanied impaired skeletal muscle performance, while treatment with metformin and JTXK granules prevented this alteration (Figure 1(i), *P* < 0.05). Next, we performed H&E staining and electron microscopy of skeletal muscle tissue (Figures 1(g) and 1(h)). Skeletal muscle fibers of diabetic mice were arranged irregularly with inflammatory infiltration observed. There were lipid droplets and swelling mitochondria in the skeletal muscle tissue, while these pathological changes were ameliorated in Met and JTXK groups. Thus, we concluded from the above results that JTXK granules attenuated body weight gain, reduced body fat mass, improved body lean mass, and enhanced muscle performance of diabetic mice.

### 3.2. JTXK Granules Improved Glucose Tolerance and Insulin Sensitivity and Decreased Lipid Content of Diabetic Mice

**Figure 2:**
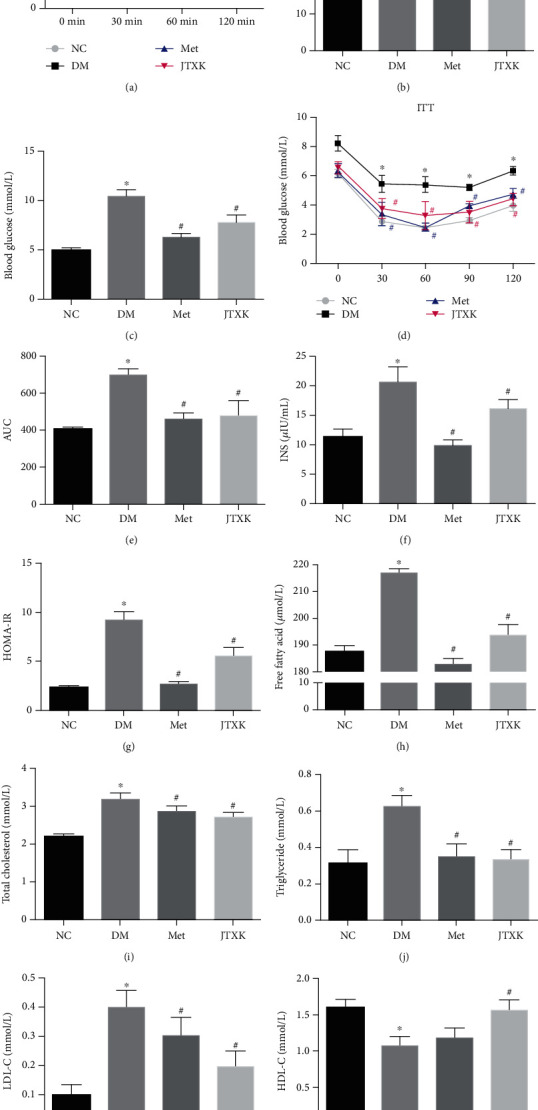
JTXK granules improved glucose tolerance, insulin sensitivity, and lipid profiles in diabetic mice. (a) OGTT test results and (b) area under the curve. (c) Fasting blood glucose. (d) ITT test and (e) area under the curve. (f) Fasting serum insulin level. (g) Homeostasis model assessment: insulin resistance in each group after treatment. Lipid profiles are represented by serum (h) free fatty acid, (i) total cholesterol, (j) triglyceride, (k) LDL-C, and (l) HDL-C contents. NC, DM, Met, and JTXK mean the normal control, diabetes, metformin, and Jiang Tang Xiao Ke granule groups, respectively. Data were presented as mean ± SD. *n* = 8 in each group. ^∗^*P* < 0.05, compared with the NC group. ^#^*P* < 0.05, compared with the DM group.

To investigate the effect of JTXK granules on glucose tolerance and insulin sensitivity, OGTT and ITT were conducted after 8 weeks of treatment. Blood samples were collected for detection of serum glucose, insulin levels, and lipid contents. Compared with normal mice, mice in the DM group showed evidently impaired glucose tolerance with obvious hyperglycemia before and after oral administration of glucose (Figure 2(a), *P* < 0.05). The area under the curve (AUC) among groups was 14.28 ± 4.17 (NC), 27.57 ± 8.19 (DM), 14.57 ± 2.45 (Met), and 14.53 ± 3.96 (JTXK), which indicated that treatment with metformin and JTXK granules improved glucose tolerance significantly (Figure 2(b), *P* < 0.05). Consistent with OGTT, results from serum glucose also revealed that diabetic mice exhibited apparent hyperglycemia, while metformin and JTXK granules could improve glucose metabolism (Figure 2(c), *P* < 0.05). In addition, long-time HFD feeding also leads to reduced insulin sensitivity. The results of ITT showed that the blood glucose level of diabetic mice was still higher than that of the other mice 30, 60, 90, and 120 min after insulin injection intraperitoneally. And AUC of ITT in the DM group (700.90 ± 61.85) was evidently increased when compared with that in the NC group (403.50 ± 28.43). Notably, metformin and JTXK granule treatment decreased blood glucose levels at 30, 60, 90, and 120 min and decreased AUC of ITT significantly (Figures 2(d) and 2(e), *P* < 0.05). Moreover, treatment with metformin and JTXK granules also attenuated the HFD-induced hyperinsulinemia and abnormal HOMA-IR index markedly (Figures 2(f) and 2(g), *P* < 0.05). As for blood lipid profiles, long-time HFD feeding caused dyslipidemia in diabetic mice, represented by the ascended FFA, TC, TG, and LDL-C level and descended HDL-C level (Figures 2(h)–2(l), *P* < 0.05). Aside from HDL-C, metformin and JTXK granule treatment markedly attenuated these alterations (Figures 2(h)–2(k), *P* < 0.05). Compared with diabetic mice, JTXK mice also exhibited a higher level of serum HDL-C (Figure 2(l), *P* < 0.05). In brief, JTXK granule treatment improved glucose metabolism and insulin sensitivity and partly reversed abnormal serum lipid levels.

### 3.3. JTXK Granules Activated AMPK/SIRT1/PGC-1*α* Signaling in Skeletal Muscle of Diabetic Mice

**Figure 3:**
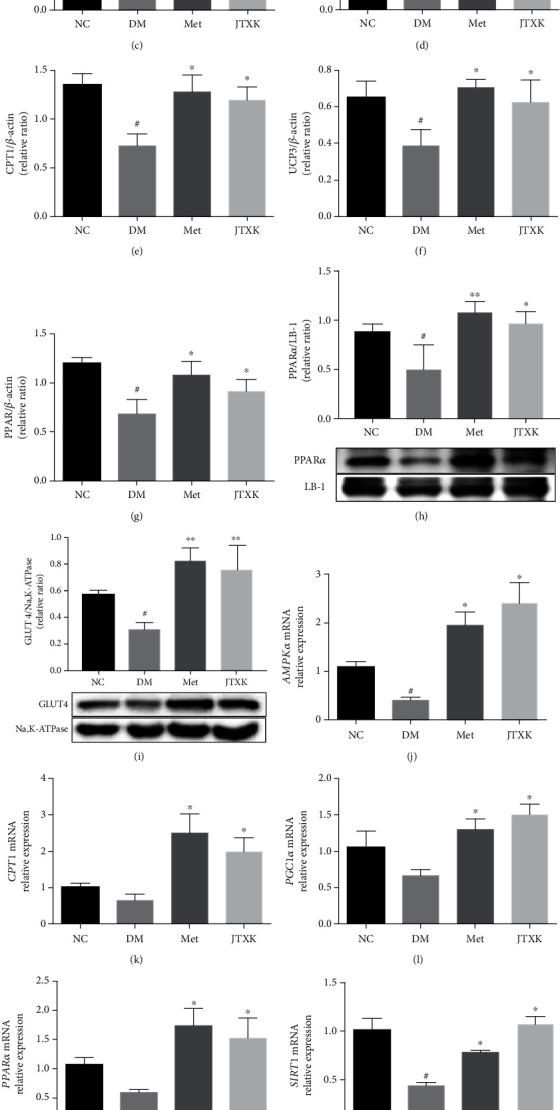
Effect of JTXK granules on AMPK/SIRT1/PGC-1*α* signaling in skeletal muscle of HFD-induced diabetic mice. The relative protein expression levels of AMPK*α*, SIRT1, PGC-1*α*, CPT1, UCP3, and PPAR*α* (both in cytosolic and in nuclear) in the skeletal muscle (a–h) and GLUT4 membrane translocation (i) were determined by western blotting. Gene expressions of AMPK*α*, CPT1, PGC-1*α*, PPAR*α*, SIRT1, and UCP3 (j–o) were determined by RT-PCR. NC, DM, Met, and JTXK refer to the normal control, diabetes, metformin, and Jiang Tang Xiao Ke granule groups, respectively. Data were presented as mean ± SD. *n* = 6 in each group. ^∗^*P* < 0.05, compared with the NC group. ^#^*P* < 0.05, compared with the DM group.

To explore the underlying mechanism of JTXK granules in ameliorating skeletal muscle IR of diabetic mice, we analyzed the expression of important proteins and genes in the AMPK/SIRT1/PGC-1*α* signaling pathway. As shown in Figures 3(a)–3(h), the protein expressions of AMPK*α*, SIRT1, PGC-1*α*, PPAR*α* (both in cytosolic and in nuclear), CPT1, and UCP3 in skeletal muscle of diabetic mice were evidently lower than that of normal mice (*P* < 0.05). After 8 weeks of intervention with metformin or JTXK granules, expressions of those proteins in these two groups were significantly increased in comparison with that in diabetic mice (*P* < 0.05). Diabetic mice also exhibited reduction of GLUT4 membrane translocation, while treatment with metformin and JTXK granules increased GLUT4 translocation (Figure 3(i), *P* < 0.05). As shown in Figures 3(j)–3(o), the mRNA expressions of AMPK*α*, SIRT1, and UCP3 in skeletal muscle of diabetic mice were lower than that of normal mice (*P* < 0.05), while treatment with metformin or JTXK granules markedly increased these gene expressions by approximately 2-fold (*P* < 0.05). Compared with the DM group, metformin or JTXK granules also increased PGC-1*α*, PPAR*α*, and SIRT1 expression significantly (*P* < 0.05). The above results indicated it is possible that JTXK granules ameliorated skeletal muscle IR through modulation of the AMPK/SIRT1/PGC-1*α* pathway.

### 3.4. JTXK Granules Improved Glucose Uptake and Mitochondrial Respiration in C2C12 Cells via Activation of AMPK*α*

**Figure 4:**
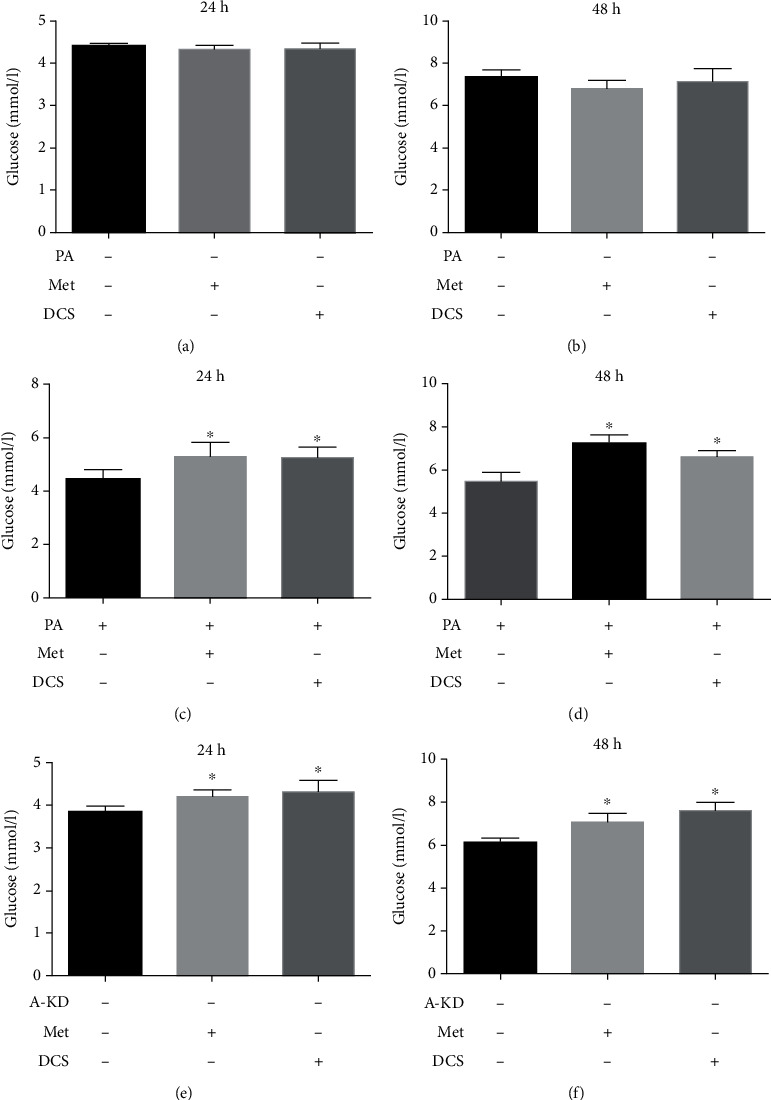
Effect of JTXK granule drug-containing serum on glucose uptake of C2C12 cells. Glucose uptake ability after intervention with drug-containing serum of JTXK granules in (a, b) normal C2C12 cells, (c, d) IR C2C12 cells, and (e, f) AMPK*α* knockdown C2C12 cells. A-KD indicates AMPK*α* knockdown. PA, Met, and DCS mean palmitic acid, metformin, and drug-containing serum of Jiang Tang Xiao Ke granules, respectively. Data were presented as mean ± SD. ^∗^*P* < 0.05, compared with the blank control.

To further verify the effect of JTXK granules through in vitro experiment, we analyzed the glucose uptake ability and mitochondrial respiration in C2C12 cells after intervening with DCS of JTXK or metformin. As shown in Figures 4(a) and 4(b), for normal differentiated C2C12 cells, intervention with metformin or DCS did not affect their glucose uptake ability (*P* > 0.05). Next, we induced C2C12 cell insulin resistance by exposing them to 0.4 M palmitic acid for 24 h. According to the results, IR C2C12 cells exhibited impaired glucose uptake capacity, while treatment with metformin or DCS for 24 or 48 h significantly improved the glucose consumption of IR C2C12 cells (Figures 4(c) and 4(d), *P* < 0.05). In the in vivo experiment, obvious alteration in the AMPK/SIRT1/PGC-1*α* pathway was observed, so we assumed that JTXK probably acts through this signaling pathway. Therefore, we next analyzed the glucose uptake ability in the AMPK*α* knockdown C2C12 cell line (A-KD group in the figure). As shown in Figures 4(e) and 4(f), after 24 and 48 h of intervention with metformin or DCS, glucose consumption in these two groups was apparently enhanced (*P* < 0.05).

**Figure 5:**
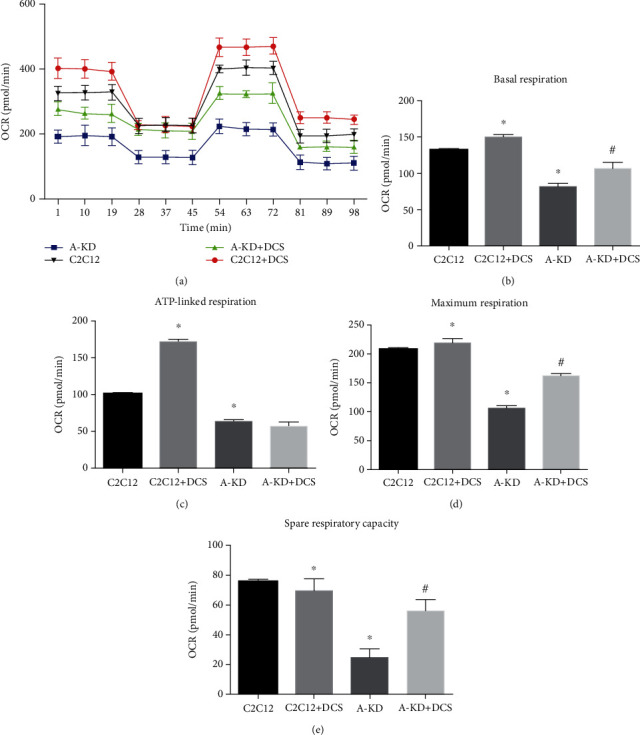
Effect of drug-containing serum of JTXK granules on mitochondrial respiration of C2C12 cells. (a) Oxygen consumption rate, (b) basal respiration, (c) ATP-linked respiration, (d) maximum respiration, and (e) spare respiratory capacity measured by Seahorse. A-KD and DCS indicate AMPK*α* knockdown and drug-containing serum of Jiang Tang Xiao Ke granules, respectively. Data were presented as mean ± SD. ^∗^*P* < 0.05, compared with the C2C12 group. ^#^*P* < 0.05, compared with the A-KD group.

As AMPK is the pivot regulator in energy metabolism, we further investigated the effects of JTXK on mitochondrial energy metabolism (Figure 5(a)). Compared with the control group, DCS of JTXK granules increased the basal respiration (Figure 5(b)), ATP-linked respiration (Figure 5(c)), maximum respiration (Figure 5(d)), and spare respiratory capacity (Figure 5(e)) of C2C12 cells with statistical significance (*P* < 0.05). For the AMPK*α* knockdown C2C12 cell line (A-KD group), compared with C2C12 cells, the basal respiration, ATP-linked respiration, maximum respiration, and spare respiratory capacity of C2C12 cells were significantly decreased (*P* < 0.05). As expected, the impaired mitochondrial respiration of AMPK*α* knockdown C2C12 cells was restored by intervention with DCS (*P* < 0.05). Furthermore, the effect of DCS on A-KD C2C12 cells was weaker than that on C2C12 cells, indicating that the effect of DCS on improving mitochondrial energy metabolism of C2C12 cells is closely related to the regulation of AMPK*α*. Thus, we concluded that AMPK*α* was closely involved in the effect of JTXK on enhancing glucose uptake and mitochondrial respiration in C2C12 cells.

### 3.5. JTXK Granules Promoted AMPK/SIRT1/PGC-1*α* Signaling in IR C2C12 Cells

**Figure 6:**
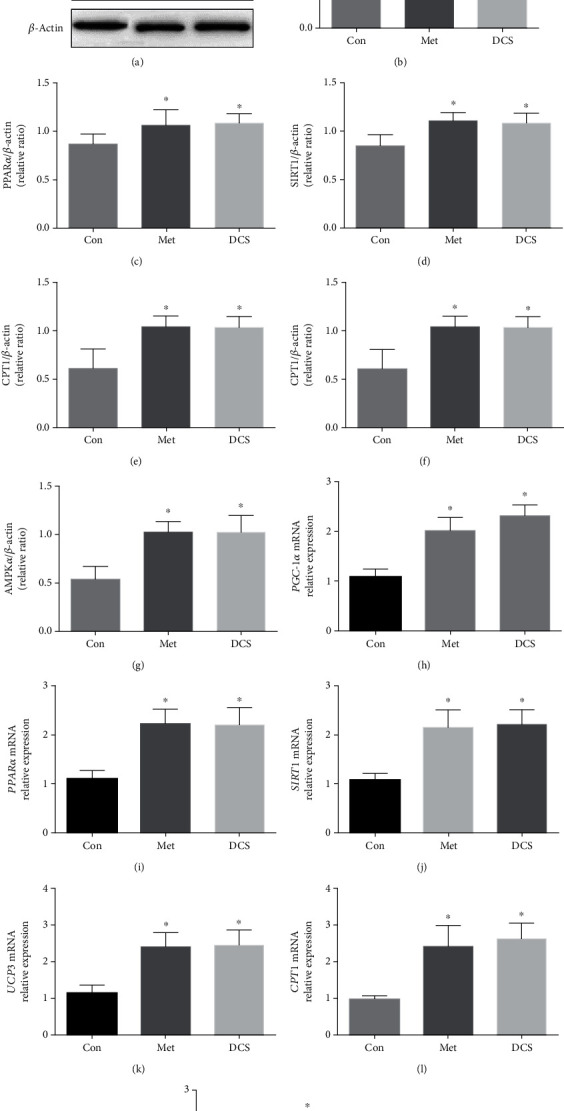
Effect of JTXK granule drug-containing serum on AMPK/SIRT1/PGC-1*α* signaling in IR C2C12 cells. (a–g) Protein expressions of AMPK*α*, SIRT1, PGC-1*α*, PPAR*α*, UCP3, and CPT1 measured by western blotting. (h–m) Relative gene expressions of AMPK*α*, SIRT1, PGC-1*α*, PPAR*α*, UCP3, and CPT1 measured by RT-PCR. Con, Met, and DCS mean the control, metformin, and drug-containing serum of Jiang Tang Xiao Ke granules, respectively. Data were presented as mean ± SD. ^∗^*P* < 0.05, compared with the Con group.

Next, we further investigated whether DCS of JTXK could affect AMPK/SIRT1/PGC-1*α* signaling in C2C12 cells. As shown in Figures 6(a)–6(g), DCS of JTXK markedly upregulated the protein expressions of AMPK*α*, SIRT1, PGC-1*α*, PPAR*α*, UCP3, and CPT1 (*P* < 0.05). Consistent with results of western blotting, DCS of JTXK increased AMPK*α*, SIRT1, PGC-1*α*, PPAR*α*, UCP3, and CPT1 mRNA expressions by 1.87-, 2.09-, 2.17-, 2.03-, 2.19-, and 2.62-fold (*P* < 0.05, Figures 6(h)–6(m)). Therefore, it is reasonable to postulate that JTXK granules could promote AMPK/SIRT1/PGC-1*α* signaling in IR C2C12 cells.

## 4. Discussion

The present study demonstrated the following. (1) JTXK granules attenuated body weight gain, reduced body fat mass, improved body lean mass, and enhanced muscle performance of diabetic mice. (2) JTXK granules improved glucose metabolism and insulin sensitivity and partly reversed abnormal serum lipid levels. (3) JTXK granules increased the gene as well as protein expression of important molecules in the AMPK/SIRT1/PGC-1*α* pathway, both in skeletal muscle tissue of HFD-fed mice and in C2C12 cells. (4) The JTXK granule was capable of enhancing glucose uptake and mitochondrial respiration in C2C12 cells, and AMPK*α* was proven to be closely involved in this process.

In the last decades, significant investigation breakthroughs have been witnessed in the study of traditional Chinese medicine. Among these, herbal medicine stands in a critical position in traditional Chinese medicine practice. The JTXK granule is an effective herbal formula derived from rich clinical practice and exhibits a satisfying therapeutic effect in the management of IR-related metabolic disorders, including obesity, prediabetes, and overt diabetes. This formula is composed of *Rehmannia glutinosa* (Gaertn.) DC. (Dihuang), *Panax ginseng* C.A.Mey. (Renshen), *Salvia miltiorrhiza* Bunge (Danshen), *Cornus officinalis* Siebold & Zucc. (Shanzhuyu), *Coptis chinensis* Franch. (Huanglian), *Pueraria montana* var. lobata (Gegen), and so on. To better understand the essential and substantial basis of this formula, the fingerprint technique was applied to illustrate the main ingredients (Fig S1). According to our previous studies, JTXK granules ameliorated IR in diabetic mice by regulating PI3K/Akt signaling in skeletal muscles [17]. Besides, we also found that ginsenoside Rb1, one of the ingredients recognized in JTXK granules, exerts a beneficial effect on increasing insulin sensitivity in skeletal muscle of obese mice. And the mechanism is through upregulating AMPK*α* expression and increasing AMPK phosphorylation [20]. Here, we proved a novel mechanism of JTXK granules in mitigation of skeletal muscle IR through activation and modulation of AMPK/SIRT1/PGC-1*α* signaling concerning mitochondrial respiration.

Western diet and sedentary lifestyle have made metabolic disorders such as diabetes, obesity, and metabolic syndrome become an important disease spectrum threatening human health. Among metabolic disorders, IR is a common phenomenon as well as the main pathological factor. Therefore, investigating effective drug candidates for IR has been a hot topic in this research field. In the present study, we chose metformin, the first-line drug for diabetes, as the positive control to compare the effect of JTXK granules on skeletal muscle IR. In line with our previous results, JTXK granules exhibited a favorable effect on attenuating body weight gain and reducing food intake. In addition, JTXK granules also helped to regulate body composition, ameliorate pathological changes in skeletal muscle, and enhance muscle performance. In the skeletal muscle of diabetic mice, we also observed lipid droplets and swelling mitochondria, and JTXK granule intervention obviously relieved these changes. Studies have shown that compared with insulin-sensitive individuals, the mitochondrial DNA copy number in leukocytes of obese people was 6.9-fold lower, and the number of mitochondria was positively correlated with glucose utilization and oxidation while negatively correlated with BMI and serum FFA levels [5, 23]. Moreover, overnutrition (e.g., HFD) results in an elevated lipid burden (e.g., FFA) as well as mitochondrial oxidative stress, which will lead to the accumulation of incomplete fatty acid oxidation and reactive oxygen species (ROS). Both incomplete fatty acid oxidation and ROS contribute to impairment in insulin action and are considered closely related to the development of IR [24].

Although the exact mechanism that leads to the development of IR in skeletal muscle is not yet fully understood, accumulation of glucose and FFA have been shown to play a primary role [25, 26]. In the current study, we observed impaired glucose tolerance and abnormal lipid profiles in diabetic mice. After 8 weeks of treatment with JTXK granules, fasting blood glucose and insulin levels were significantly decreased, along with improved glucose tolerance and insulin sensitivity. JTXK granule intervention also reduced serum FFA, TC, TG, and LDL-C contents and increased HDL-C content significantly. As mentioned before, elevated glucose and FFA levels were the main cause of glucolipotoxicity [27]. And the consequences of glucolipotoxicity involve mitochondrial dysfunction, ROS production, and endoplasmic reticulum stress, of which all contribute to the progression of metabolic disorders [28]. This raises the possibility that JTXK granules might act through the signaling pathway that is related to mitochondrial biogenesis or metabolism.

In the present study, we claimed that JTXK granules enhanced expression levels of proteins and genes related to mitochondrial energy metabolism, including AMPK*α*, PGC-1*α*, SIRT1, PPAR*α*, UCP3, and CPT1. JTXK granules also enhanced GLUT4 membrane translocation, which is considered a primary mediator of glucose removal from the blood circulation as well as an essential regulator of whole-body glucose homeostasis [29]. PGC-1*α* emerged as a key transcriptional coactivator in understanding how nuclear regulatory signals are linked to the biogenesis of mitochondria, antioxidant defense, and inflammatory response in skeletal muscle [30]. Accumulating studies have shown that HFD induces downregulation of PGC-1*α*, while its overexpression prevents HFD-induced reduction of mitochondrial respiration in hepatocytes and promotes exercise-induced autophagy in skeletal muscle [31, 32]. As the most prominent and extensively studied member of sirtuins, SIRT1 plays a critical role in the regulation of PGC-1*α* by deacetylation [33]. Besides, SIRT1 and PPARs both trigger mitochondrial biogenesis, so activation of PGC-1*α*/PPARs might offer a novel strategy in the treatment of mitochondrial-related diseases [34]. AMPK and SIRT1 are both upstream regulatory factors of PGC-1*α*. There may be an internal relationship between them, which plays a significant role in energy metabolism. After AMPK activation, it acts on the downstream SIRT1 by affecting the NAD^+^ level, which enhances mitochondrial oxidative phosphorylation capacity and ATP production through the SIRT1/PGC-1*α* signaling pathway [35]. So it is clear that the AMPK/SIRT1/PGC-1*α* network is a critical energy-sensing signaling pathway and that AMPK activation will induce the concurrent deacetylation and phosphorylation of its downstream targets and relieve the susceptibility to IR-associated metabolic disorders [36]. It reveals that AMPK activation helps prevent diabetic disorders in many tissues and organs, including skeletal muscle, hepatic tissue, and renal podocytes, by suppressing apoptosis and reducing oxidative stress [37–39]. To the best of our expectation, AMPK*α* knockdown C2C12 cells exhibited apparent impaired mitochondrial respiration capacity, indicating the essential position of AMPK*α* in mitochondrial metabolism. JTXK granules restored mitochondrial respiration capacity in IR C2C12 cells but were less effective on A-KD C2C12 cells, which proved that AMPK*α* regulation was closely involved in the therapeutic mechanisms of JTXK granules. Taken together, our findings provide us a novel image that the JTXK granule ameliorates skeletal muscle IR through activation of AMPK*α* and modulation of the AMPK/SIRT1/PGC-1*α* signaling network.

## 5. Conclusions

In sum, we demonstrate that JTXK granules could improve glucose and lipid metabolism and ameliorate skeletal muscle IR, which are associated with activation of the AMPK/SIRT1/PGC-1*α* signaling pathway (Fig S2). Our results indicate that the JTXK granule is a promising formula to be promoted in clinical use for the treatment of obesity, T2DM, and other IR-related metabolic disorders. However, further strictly designed clinical trials for the clinical promotion of JTXK are still necessary. And caution must be taken when considering the study duration, intervention methods, dosage and drug forms, inclusion criteria, and so on, for every single element counts when evaluating the therapeutic effect of the Chinese herbal medicine formula.

## Figures and Tables

**Table 1:** Sequences of RT-PCR primers.

Gene	Primer sequences (5′-3′)
AMPK*α*	F: AAACCCACAGAAATCCAAACAC
	R: CCTTCCATTCATAGTCCAACTG
PGC-1*α*	F: CCCTGCCATTGTTAAGACC
	R: TGCTGCTGTTCCTGCTCCT
SIRT1	F: TTGTGAAGCTGTTCGTGGAG
	R: GGCGTGGAGGTTTTTCAGTA
PPAR*α*	F: AGGAAGCCGTTCTGTGACAT
	R: TTGAAGGAGCTTTGGGAAGA
CPT1	F: AGAACCACCAAAGCGGAAA
	R: TCCCACAGGAGACAGAAACC
UCP3	F: CCCTGACTCCTTCCTCCCTG
	R: GCACTGCAGCCTGTTTTGCTGA

## Data Availability

The original data will be available upon request.

## Additional Points

*Highlights*. JTXK granules attenuated body weight gain and improved glucolipid metabolism of diabetic mice. JTXK granules improved skeletal muscle insulin sensitivity of diabetic mice. JTXK granules improved glucose uptake and mitochondrial respiration via activation of AMPK*α*. JTXK granules ameliorated diabetes through activating AMPK/SIRT1/PGC-1*α* signaling.

## Supplementary Materials

Supplementary 1 Fig S1: fingerprint chromatogram of JTXK granules. (10) Puerarin. (16) Coptisine. (18) Salvia acid B. (19) Berberine. (22) Paeonol.

Supplementary 2 Fig S2: graphic abstract. JTXK granules improved glucolipid metabolism and ameliorated skeletal muscle IR through the regulation of the AMPK/SIRT1/PGC-1*α* signaling pathway.
