# Supplementary figures and images for: Protective Effect of Jiang Tang Xiao Ke Granules against Skeletal Muscle IR via Activation of the AMPK/SIRT1/PGC-1α Signaling Pathway
Source: Oxid Med Cell Longev. 2021 Jul 3;2021:5566053. doi: 10.1155/2021/5566053 (PMC8277912; doi:10.1155/2021/5566053)

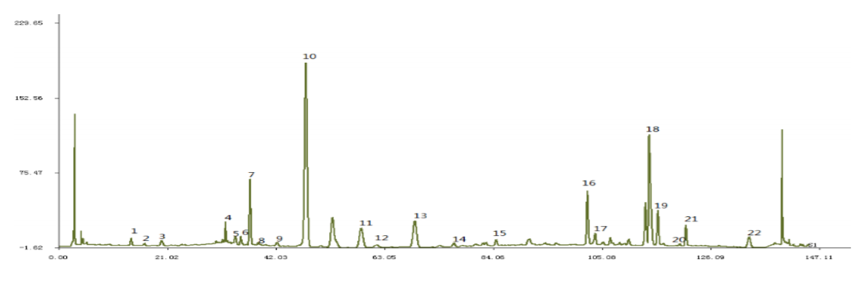

Supplement: Supplementary 1 — Fig S1: fingerprint chromatogram of JTXK granules. (10) Puerarin. (16) Coptisine. (18) Salvia acid B. (19) Berberine. (22) Paeonol. [file 5566053.f1.png]

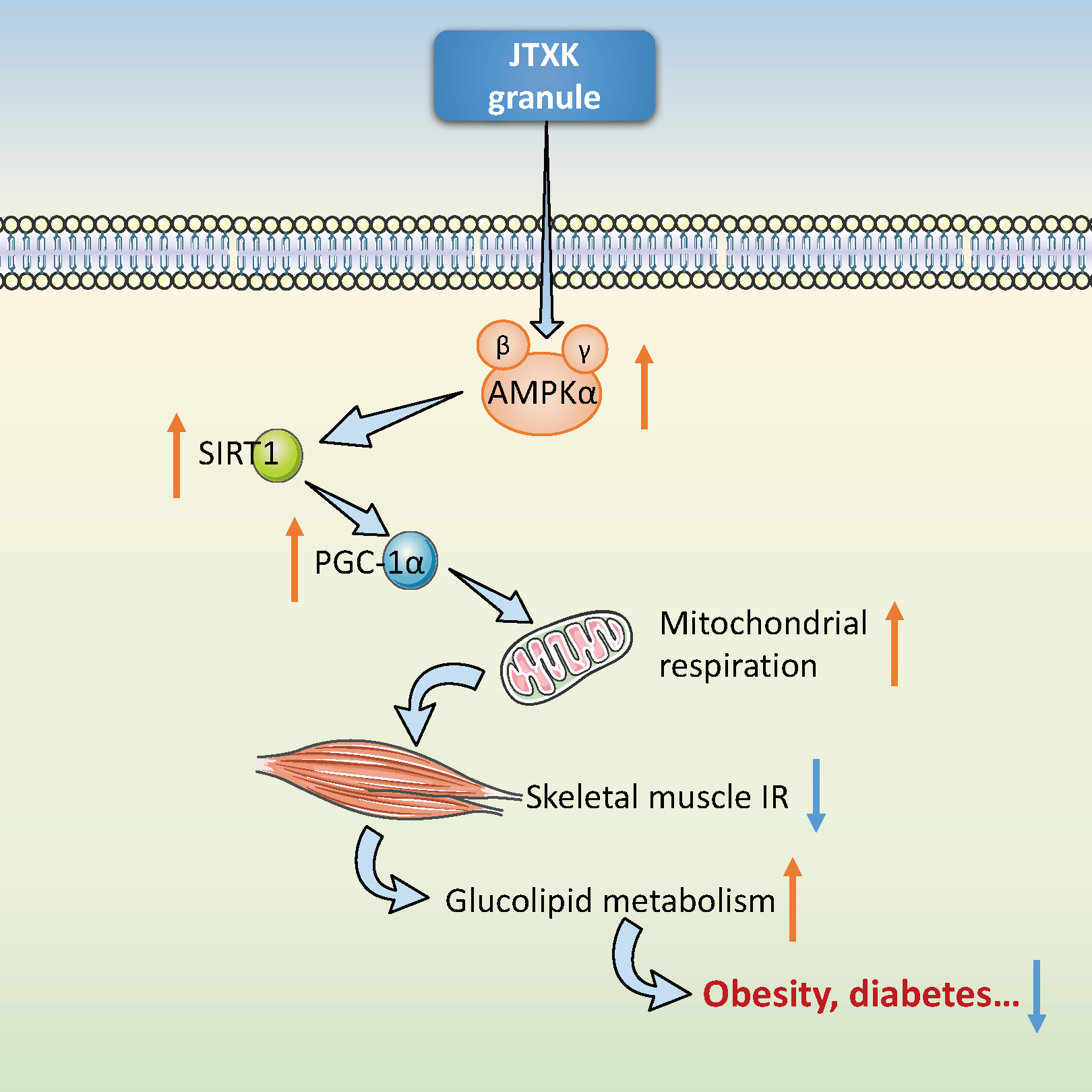

Supplement: Supplementary 2 — Fig S2: graphic abstract. JTXK granules improved glucolipid metabolism and ameliorated skeletal muscle IR through the regulation of the AMPK/SIRT1/PGC-1α signaling pathway. [file 5566053.f2.png]
